# Wideband end-fire antenna based on modulated grooved surface plasmon polaritons

**DOI:** 10.1038/s41598-025-10667-x

**Published:** 2025-07-11

**Authors:** Amitkumar Patel, Aakash Bansal, Chinthana Panagamuwa, William Whittow

**Affiliations:** Wolfson School of Mechanical, Electrical and Manufacturing Engineering, Loughborough University, Loughborough, UK

**Keywords:** Electrical and electronic engineering, Electronic and spintronic devices

## Abstract

This article presents low-profile wideband millimeter-wave (mmWave) end-fire antenna based on groundless grooved spoof surface plasmon polaritons (SSPPs) technique for applications in 5G mmWave communications. A novel linearly modulated grooved profile has been designed and optimized to convert bounded SSPP modes into radiation modes. The proposed groundless modulated grooved profile is designed to achieve a zero tilting and highly directional beam in the end-fire direction. A semi-curvilinear ground profile and stepped impedance transition profile are designed to improve impedance matching, which simultaneously enable wide bandwidth of 22 GHz to 41 GHz, i.e., fractional bandwidth (FBW) of 62% with a peak gain of 13 dBi in a low-profile structure. The overall form-factor of the antenna is 5 $\lambda _{0}$ $\times$ 0.9 $\lambda _{0}$ $\times$ 0.016$\lambda _{0}$ (where $\lambda _{0}$ is the wavelength in free space at the center frequency). The high directivity, low-profile and lightweight structure of the antenna makes it highly suitable for applications in mmWave communications.

## Introduction

Low-profile, low-cost and planar wideband end-fire antennas have been a key requirement for point-to-point wireless communication systems^1^. Classical end-fire antennas tend to have low gain^2^ and non-planar geometry^3^ making them unsuitable for compact systems such as CubeSats. SSPPs have recently been seen in the literature as an important configuration to bound the flow of electromagnetic waves between metal and dielectric material while propagating parallel to the interface and decaying exponentially in the direction perpendicular to the interface^4–6^. This type of structure exhibits low transmission loss and plays a vital role in the design of mmWave and terahertz antennas^7–9^.

**Fig. 1:**
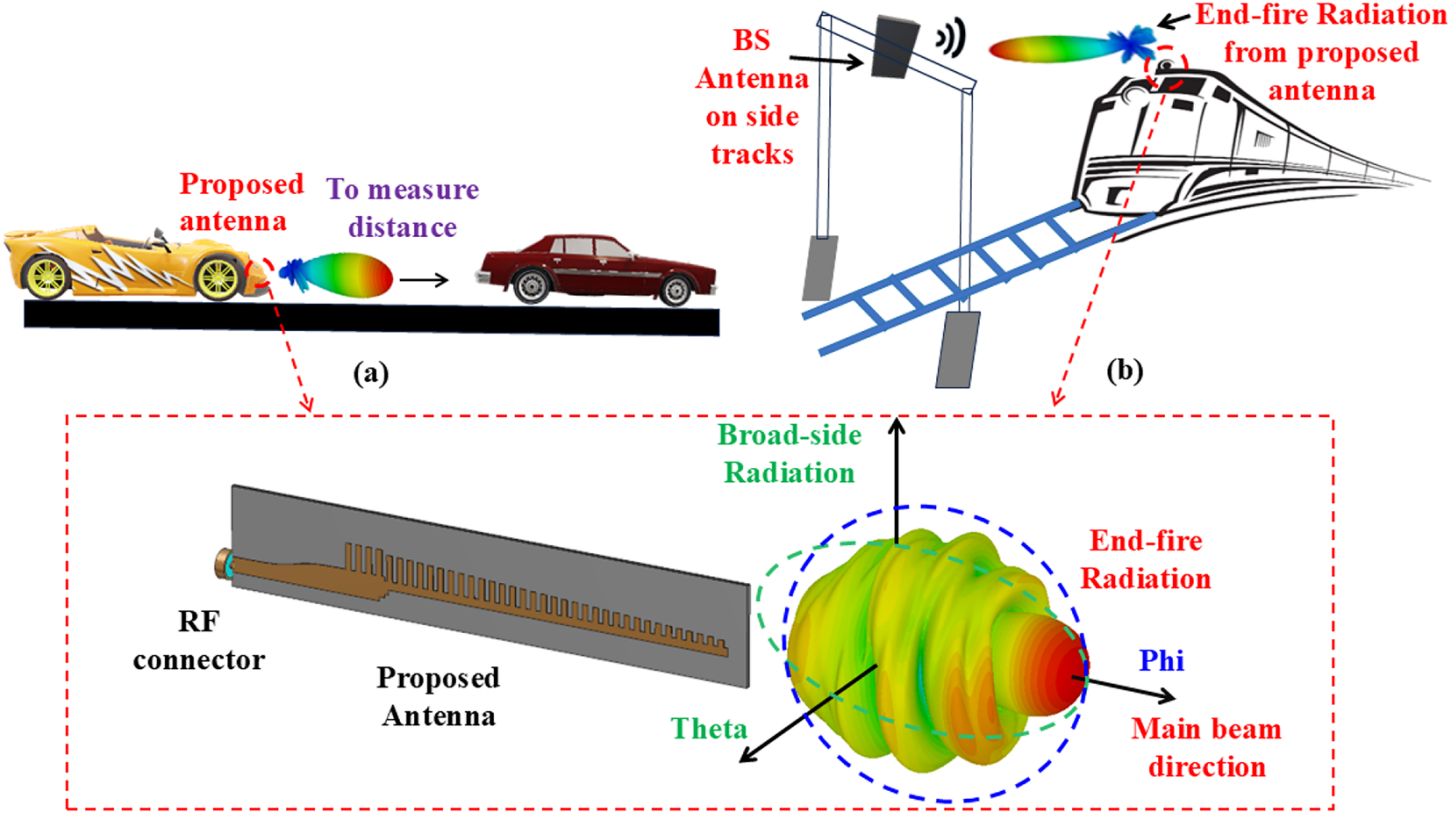
Illustration of proposed end-fire antenna applications in (**a**) automotive radar; and (**b**) high-speed train communications.

Several end-fire antennas using SSPPs are seen in the literature coupled with patches, dipoles, and Vivaldi structures to enhance bandwidth, reduce transmission loss, achieve high gain, and reduce crosstalk^7–11^. However, SSPPs in these cases are mainly used as transmission lines to feed the radiating element. Vivaldi and dipole antennas tend to have large transverse geometry making them unsuitable for beamforming array antennas^12,13^. Furthermore, a printed dipole mainly resonates at a specific designed frequency and hence, possesses a narrow impedance bandwidth (IBW). On the other hand, Vivaldi antennas achieve more than 100 % bandwidth but the main beam deviates from the end-fire direction^14^.

To mitigate these issues, gratings, 2D metallic blocks, and grooves attached to SSPPs have been tapered linearly or non-linearly enabling impedance matching and to allow the bounded modes to radiate^15–17^. An example of such a traveling-wave end-fire antenna based on groundless SSPPs is shown in^15^, where the antenna offers a peak gain of 9.2 dBi. Owing to groundless structure and strong field confinement are salient features in high-density integrated circuits and components at mmWave and Terahertz frequencies^16,17^. However, it has a narrow operating range from 7.5 to 8.5 GHz (FBW of 12.5 %) for a main beam in the end-fire direction and large sidelobe levels (SLLs)^15^. To improve the gain and efficiency of such kind structure^15^, a novel SSPPs to support odd mode proposed in^18^. It achieved a peak gain of 15 dBi and   90 % radiation efficiency in mmWave band. However,limited operating bandwidth persists. A further improvement in the performance of the end-fire antenna with SSPPs based geometry is seen in^19^, where a double-sided tapering groove has been created at both the ends of microstrip line. It used a microstrip-line to slot-line transition to feed corrugated SSPPs antenna supporting odd-mode and achieved an FBW up to 70 % with a gain of 8.6 dBi in the end-fire direction.

Most of the proposed work in the literature (as reviewed above) were carried out based on the fundamental mode supported by SSPPs. Similar structures are seen to operate with fundamental mode in corrugated substrate integrated waveguides^20–26^. However, periodically loaded SSPPs can also support the higher order modes and improved the bandwidth performance^27–31^. The principle of such structures is based on groove height variation along the length of the design, which produces a high-impedance surface. As an example,^28^ demonstrated a structure that can support the first higher-order mode to generate a peak gain of 14.3 dBi.

Another structure was reported in^29^, using tilted corrugated SSPPs and I-shaped resonators on the patch and in the ground plane to offer an FBW of 140.8 % with a peak gain of 15.2 dBi. Such an antenna needs to have a full ground plane over the corrugated SSPPs and use a linearly tapered distribution. Although this type of geometry achieved wide bandwidth and higher gain, it required double sided corrugated surfaces to generate zero tilting in end-fire direction, and extra support to hold the antenna due to thin substrate that broadened beam in E-plane.

In this article, we propose a novel low-profile, single-layer wide-band end-fire antenna for mmWave communications. Some of the applications of the proposed antenna, for instance in automotive radar and high-speed train communications, are shown in Fig. 1. The proposed geometry is derived from modulated SSPPs profile, and further optimization carried out in top-plane and ground-plane to achieve wide-band and better gain performance in low-profile. The main contributions of this proposed design are as follows:

- Wideband end-fire Radiation using SSPPs: A novel groundless, single-layer SSPP-based antenna is proposed, incorporating a linearly modulated, single-sided grooved profile that supports efficient surface plasmon propagation and guides energy in the end-fire direction consistently from 22 to 41 GHz, without beam tilt.
- High gain and efficiency: The antenna achieves a measured gain ranging from 7.5 dBi to 13 dBi, with an average gain of 10 dBi and a measured radiation efficiency exceeding 90% across the entire operating band, owing to optimized mode control and low-loss SSPP transmission.
- Compact and fabrication-friendly structure: Designed on a single-sided, groundless platform using standard photolithography, the antenna is compact (5.5 $\lambda _{0}$ $\times$ 0.1 $\lambda _{0}$ $\times$ 0.05 $\lambda _{0}$) and mechanically stable without requiring additional support foam, unlike other ultra-thin SSPP designs.
- Optimized transition and ground profile: A stepped impedance transition and semi-curvilinear slotted ground plane improve impedance matching, suppress backward radiation, and reduce side-lobe levels (SLLs), enhancing directional performance.
- Good agreement with measurement: Experimental results, including reflection coefficient, 2D radiation patterns, and gain/efficiency measurements from 24 to 40 GHz, show good agreement with simulations, validating the effectiveness of the proposed design for practical 5G mmWave applications.

The organization of the paper is as follows: “Analysis and design of grooved SSPP antenna” describes the unit-cell design of grooved SSPPs and the overall evaluation of the antenna structure; “Proposed antenna design” discusses the fabrication and measured results of the antenna followed by conclusions in “Fabrication and measurements”.

## Analysis and design of grooved SSPP antenna

This section is divided into two parts. First the grooved SSPP unit-cell is analyzed in detail, and then the antenna design is presented.

### Grooved SSPP unit-cell

To begin the design process, a unit-cell grooved SSPP is considered here to bound the fields traveling along the guiding structure and to contribute towards the radiated power. A corrugated groove (height *h* = 2.55 mm, period *p* = 1.1 mm, gap *g* = 0.5 mm, and width *w* =0.6 mm) was designed in Computer Simulation Technology (CST) Microwave Studio Software Suite 2022 to derive the dispersion curve for the 2D plasmonic waveguide structures^32^.

It is well known that metals can be considered as perfect electric conductors (PEC) at high frequencies and such groove based periodic surface support SSPPs^8^. When a quasi-transverse magnetic (TM) polarized wave is fed through the microstrip-line within the periodic SSPPs loaded surface, the wave propagates along the longitudinal direction, $\hbox {k}_{x}$, considering the operating wavelength, $\lambda \gg w$ and $\lambda \gg p$. This longitudinal direction can be obtained from the reflectance, and is defined mathematically as^29^,

1 $$\begin{aligned} k_x = k_0 \sqrt{ 1 + \frac{g^2}{p^2} \tan ^2 (k_0 h) } \end{aligned}$$

Here, $\hbox {k}_{0}$ is the wavenumber. The transverse electric field of SSPP wave decays exponentially as it moves away from the metal strip and it can be expressed as^33,34^,

2 $$\begin{aligned} E_t (x,y) = \frac{-\alpha }{j\omega \varepsilon _0} \times A e^{-\alpha y} \times e^{-\beta x} \end{aligned}$$

Here, $\alpha$ and $\beta$ represent the attenuation and phase constant of the structure, respectively. The relationship between propagation constant and attenuation constant is defined as,

3 $$\begin{aligned} k_x^2 = k_0^2 + \alpha ^2 \end{aligned}$$

One can use Eqs. (1) and (3) to derive the expression for the attenuation constant, which can be written as,

4 $$\begin{aligned} \alpha = \frac{k_0 \times w \times \tan (k_0 \times h)}{p} \end{aligned}$$

Equations (1) and (4) demonstrate that asymptotic frequencies of surface waves and the attenuation constant are primarily controlled by the height of the groove. The groove width has minimal impact on performance, as it primarily influences the propagation of higher-order modes^28^. The constantly grooved SSPP supports the single mode with better field confinement, however, it suffers from a limited operating bandwidth^28^.

**Fig. 2:**
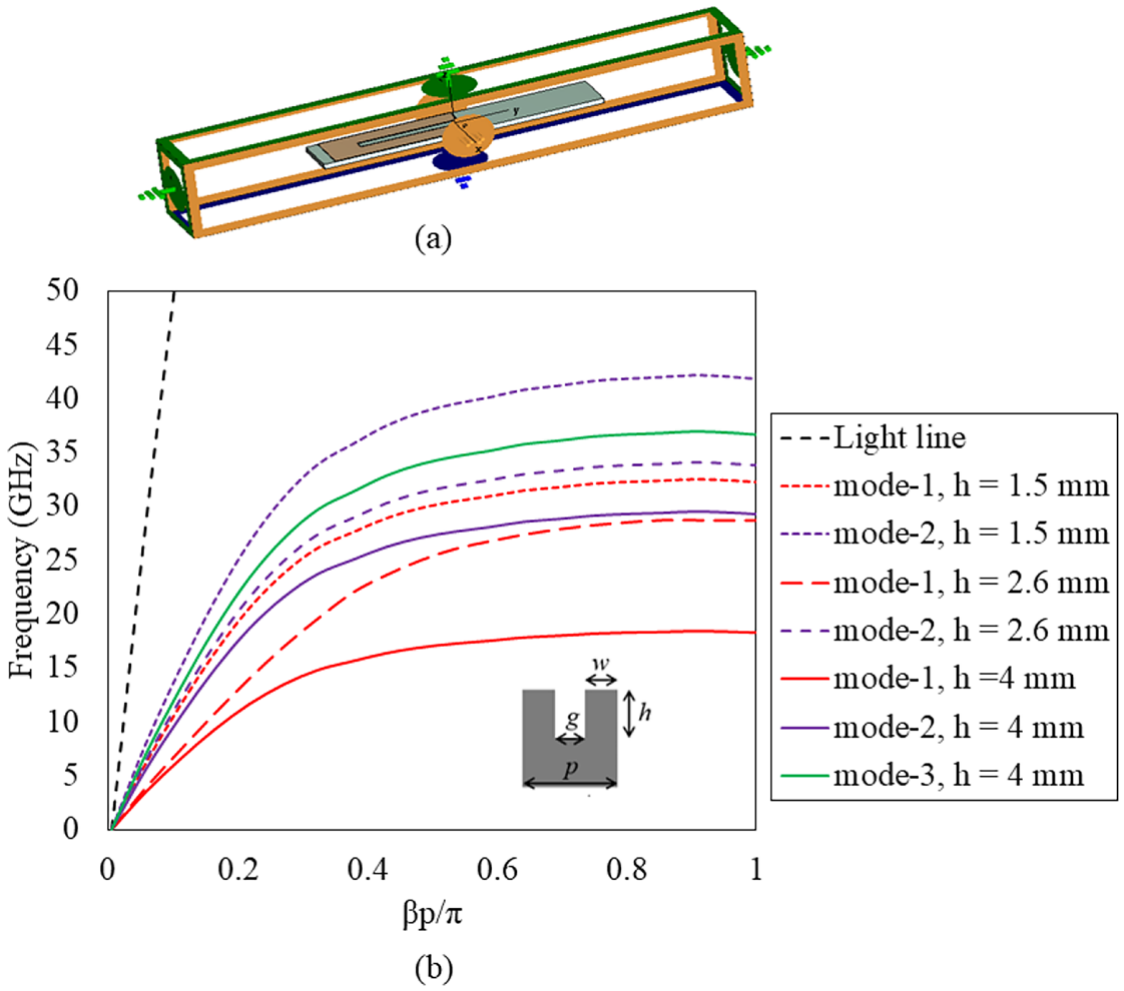
(**a**) Unit-cell design in CST; and (**b**) dispersion curve for the grooved SSPP with different groove height, *h* (inset: pictorial representation of the SSPP unit-cell).

To enable bandwidth enhancement, integration of a modulated grooved SSPP is necessary within the structure to support first as well as other higher order modes. However, higher-order modes (mode-4) exhibit weaker field confinement above the surface, as shown in Fig. 2.

As seen from Fig. 2, higher-order modes have small deviation from the light line, resulting in reduced field confinement. Field enhancement is seen to improve with higher frequency as the propagation constant diverges further from the light line, reaching its peak at the asymptotic frequency ($\hbox {k}_{x}$ = $\frac{\pi }{h}$) at the edge of the first Brillouin zone. Strong field confinement in higher-order modes occurs only when they have a lower asymptotic frequency range. Similar to the fundamental mode, the asymptotic frequency of higher-order modes can be effectively decreased by increasing the groove depth, thereby shifting each mode to a lower frequency band. Furthermore, unlike metallic waveguides and microstrip structures, the flow of each higher-order mode within the corrugated structure occupies a distinct frequency band, ensuring no overlap between modes.

The number of modes mainly depends on the height of the corrugated groove as defined in Eq. (1). For a given groove height, *h*, the supported SSPP modes number can be estimated using^28^,

5 $$\begin{aligned} N = 1 + \text {int} \left( \frac{h}{p} \right) \end{aligned}$$

When the groove height, *h* = 2.6 mm, *w* = 0.5 mm and *p* = 1.1 mm, the unit-cell supports the three modes. The phase constant of the unit-cell is just below the light line, as shown in Fig. 2. The asymptotic frequencies of mode-1, mode-2 and mode-3 are 15 GHz, 26 GHz and 46 GHz, respectively. This configuration ensures the field flow along the traveling wave direction and supports radiation in the end-fire direction.

**Fig. 3:**
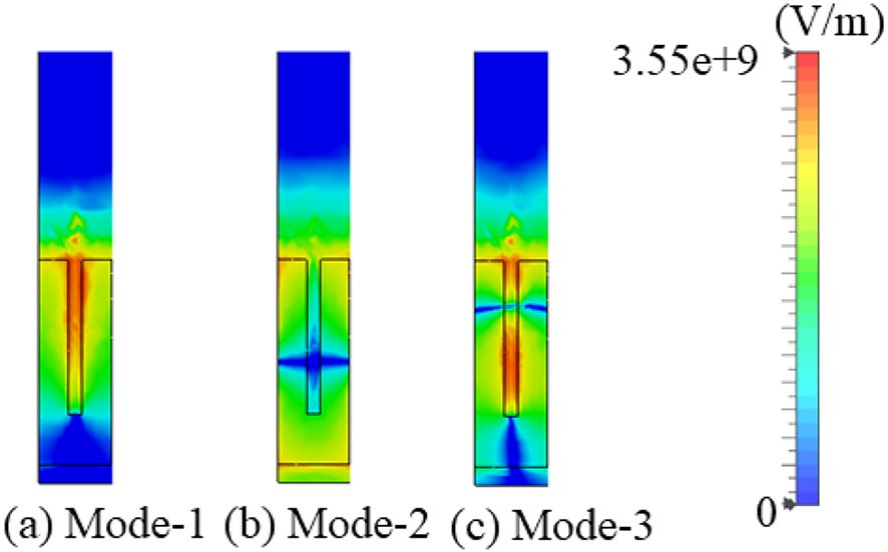
Coupling of fundamental and other higher order modes of electric fields on the SSPP unit-cell.

As the groove height increases further, the higher order mode flow at lower frequencies and their E-fields become more tightly bound within the corrugations, as illustrated in Fig. 3. However, this causes the dispersion curve to deviate significantly from the light line, resulting in reduced radiation efficiency. Conversely, smaller groove heights keep the phase constant of the structure closer to the light line. While this configuration supports a wider operating bandwidth, it comes at the cost of reduced gain and transmission efficiency. Thus, optimizing the groove height is critical to balancing bandwidth, gain, and efficiency.

Hence, a modulated profile for the grooved SSPP is needed to achieve wide operating bandwidth while maintaining transmission efficiency. Such a profile enables the support of higher-order modes by lowering their cut-off frequencies. These higher-order modes play a dominant role in enhancing the radiated power of the antenna structure, contributing to improved overall performance.

**Fig. 4:**
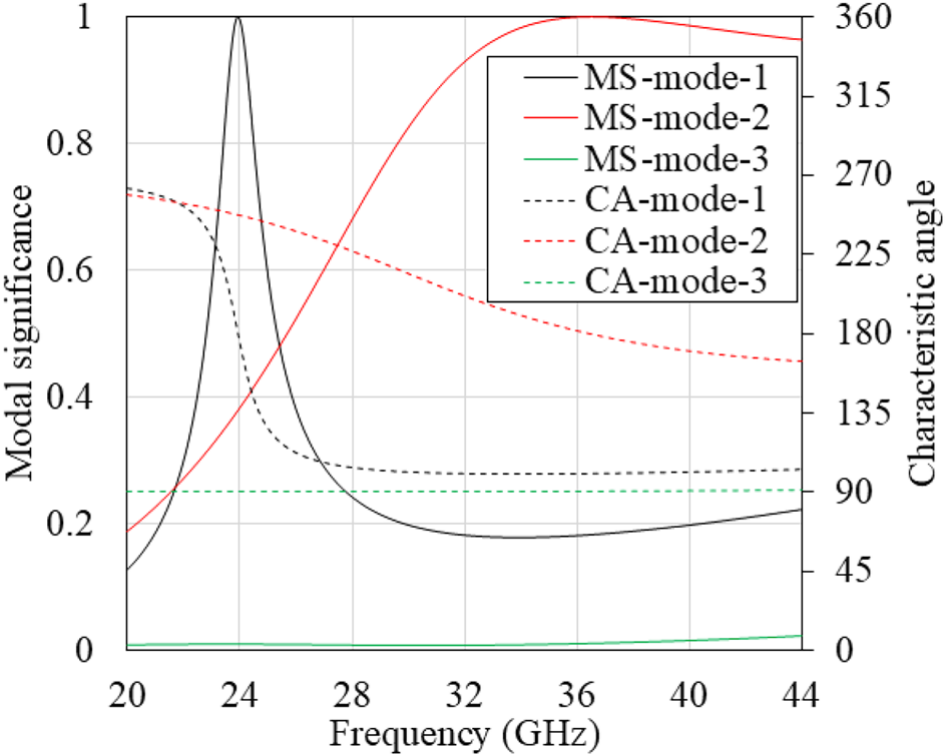
Characteristic mode analysis for a SSPP unit-cell.

To gain deeper insights into the existing modes, we performed characteristics mode analysis (CMA) of the SSPP unit-cell, focusing on modal significance (MS) and characteristic angle (CA), as shown in Fig. 4. The analytic equations for MS and CA, as presented in^35^, were employed in this analysis. MS and CA are derived from the eigenvalues and are independent of the excitation or source, providing valuable information about the modes. Figure 4 shows that characteristic mode (CM) 1 has MS $\sim$ 1 at 24 GHz hence only CM 1 will dominate in the antenna. However, it has limited operating bandwidth. On the other hand, increased frequency invited more higher order modes with large MS. Another mode that gives significant contributions from 28 to 41 GHz is CA mode 2, which can also be confirmed from the dispersion curve as well.

Another important parameter of a traveling wave antenna (TWA) is the length of the structure, which controls the peak attainable gain of the antenna. The length of the antenna can easily be derived from the Hanson-Woodyard condition^30^, and it should satisfy the following relationship,

6 $$\begin{aligned} \beta \approx k_{0} + \frac{2.94}{L} \end{aligned}$$

Here, *L* is the total length of the antenna structure. The phase constant of the surface wave above the metal grooved SSPP at 28 GHz is 750 rad/m (see Fig. 2) and can be represented as $\beta$=1.2 $\hbox {k}_{0}$. Hence, the length of the radiating part is approximated to 2 $\lambda _{0}$.

**Fig. 5:**
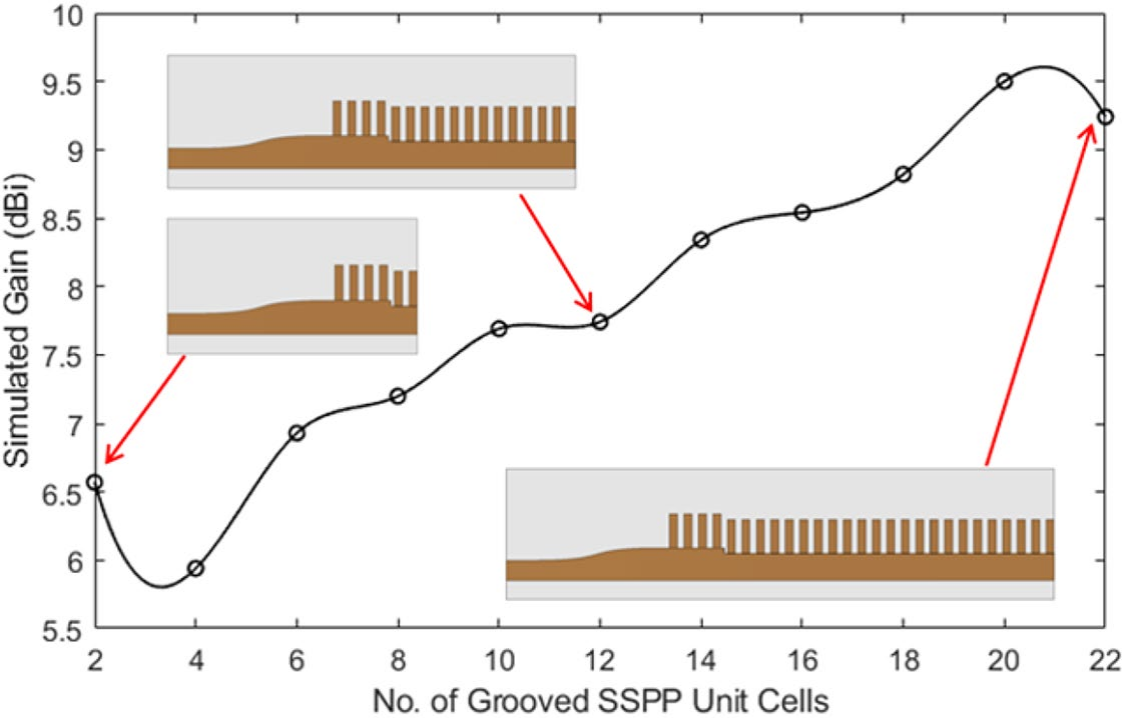
Simulated gain of the antenna as a function of number of grooves.

**Fig. 6:**
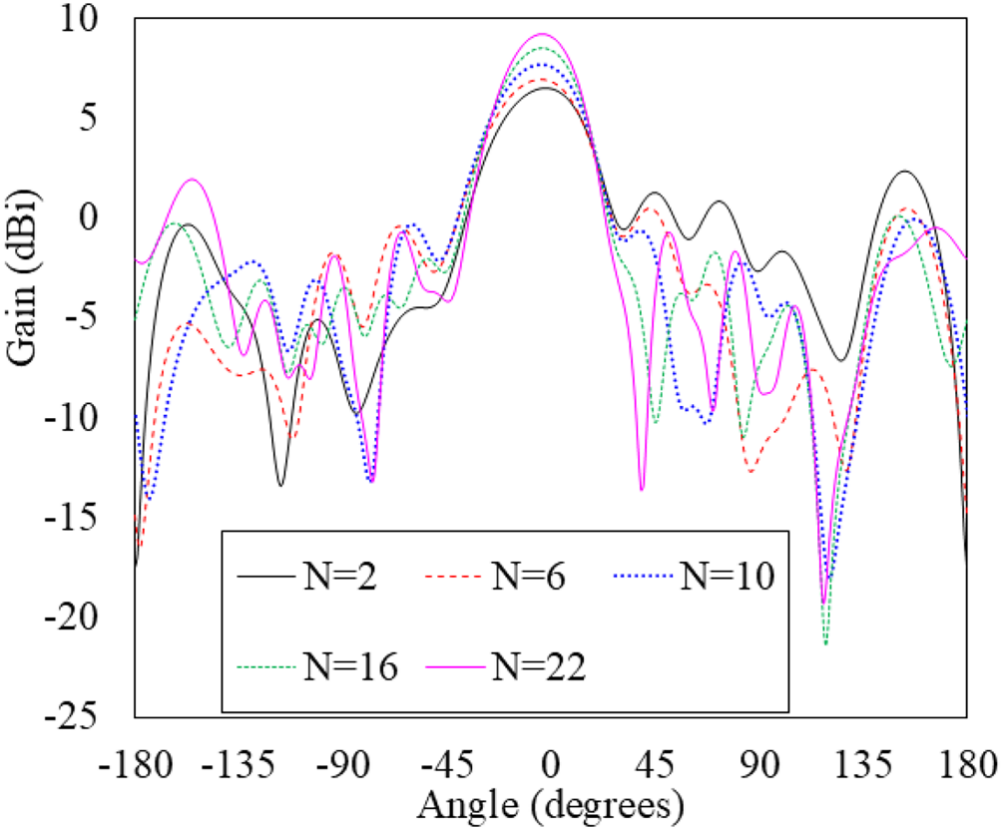
Simulated radiation patterns with varying number of grooves.

**Fig. 7:**
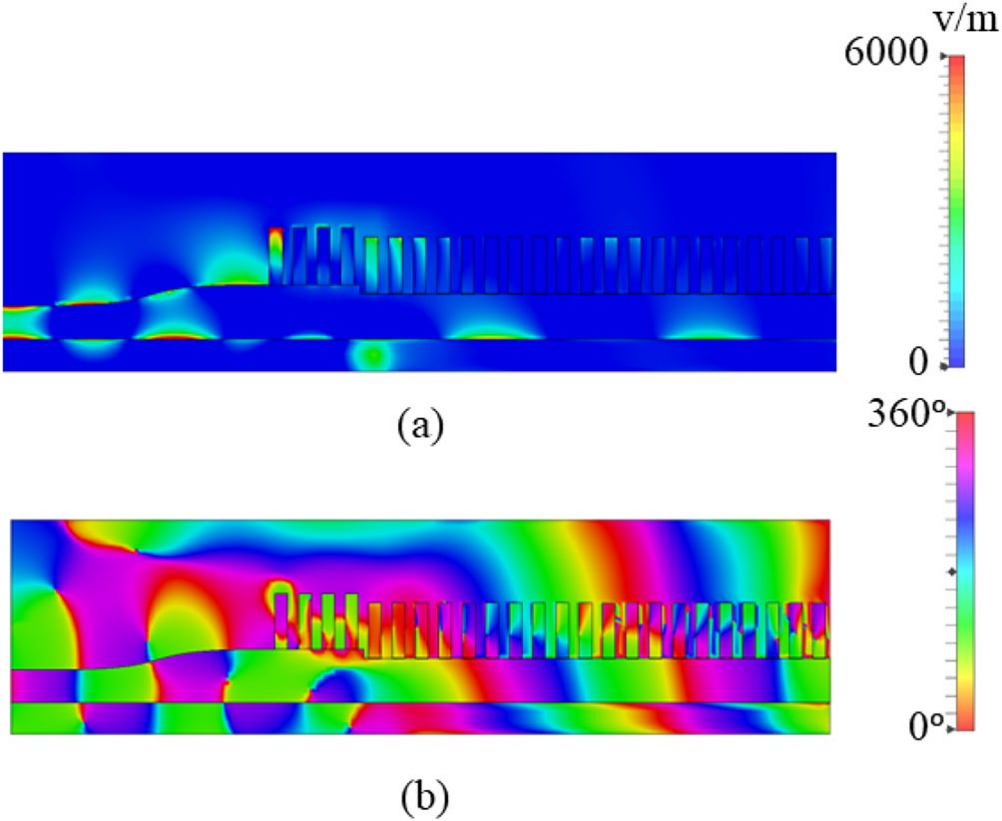
Simulated electric field distribution of the antenna with 20 grooves (a) amplitude; and (b) phase.

A parametric study of the number of grooves (and hence the length of the antenna) is conducted to see the impact on gain and radiation patterns using a commercial full wave simulator, Computer Simulation Technology (CST) Microwave Studio software, and is shown in Figs. 5 and  6, respectively. The gain of the antenna is seen to increase significantly with increase in the number of grooves up to 20. This happens because each SSPP unit contains the same amplitude and continuously variable phase of excitation, which functions as a uniform linear array. The simulated electric field’s amplitude and phase of the antenna with 20 grooves are shown in Fig. 7a, b, respectively. After 20 grooves, the antenna gain saturates and begins to decrease. Moreover, the spurious lobes emerge in the unwanted direction.

**Fig. 8:**
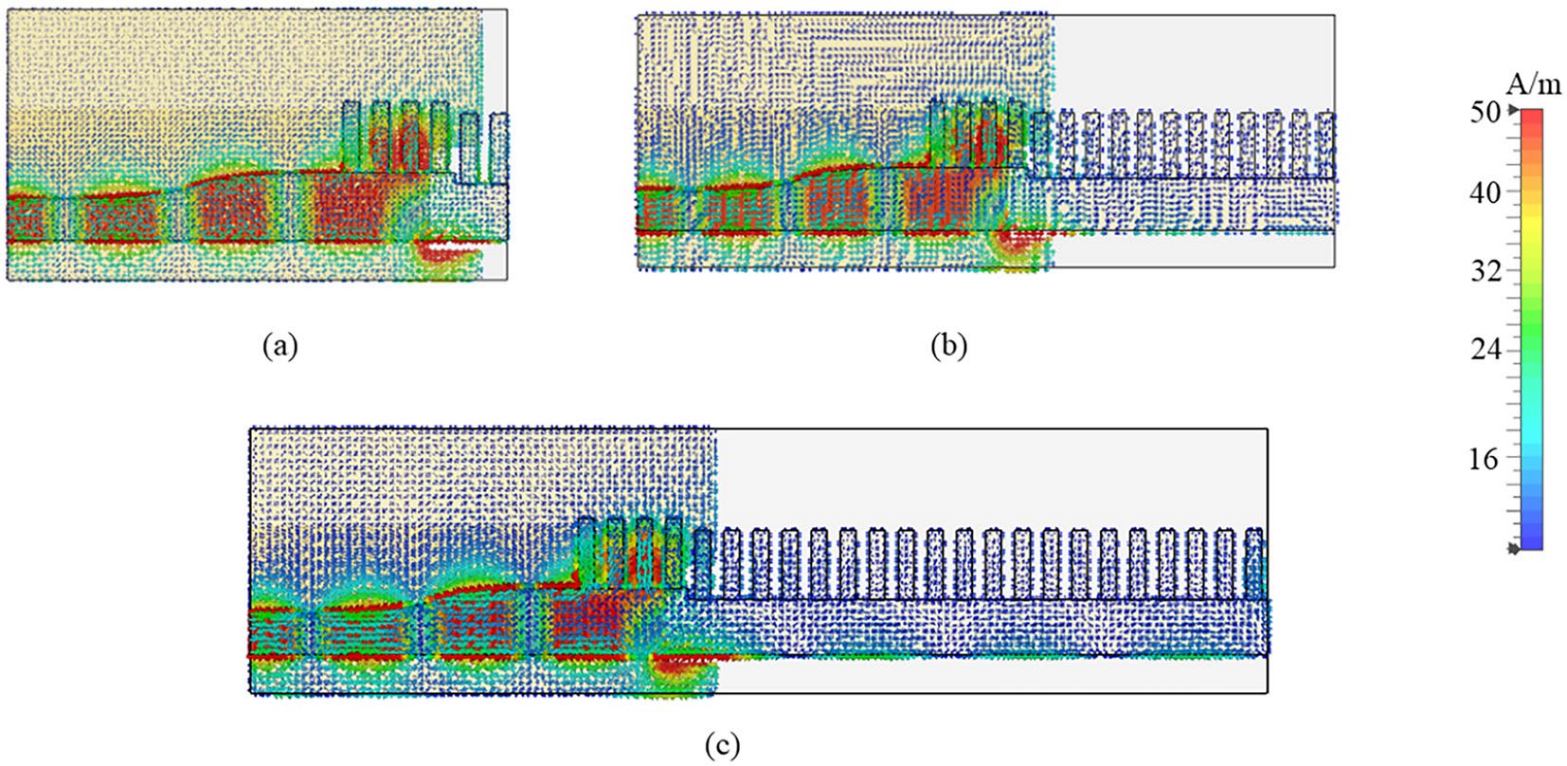
Simulated radiation patterns with varying number of grooves.

There are several causes of dropping a gain some of the primary reasons are the effect of mutual coupling between the elements that shown in Fig. 8, which can lead to destructive interference, phase errors leading to a reduction in coherent additions of signals, and the introduction of additional losses due to impedance mismatches in the elements. Furthermore, increasing the number of grooves above 16 results in higher SLLs, as shown in Figure 6. Therefore, to achieve optimal SLLs and maintain high gain, the final antenna design incorporates using 16 grooves, which is further discussed in detail in “Analysis and design of grooved SSPP antenna”(B).

At the end of the design process, it is required to match the phase constant ($\beta = \sqrt{k_{0}^2 + \alpha _{0}^2}$) of the structure with free space to ensure a better impedance match and hence, maximize the radiation^18^. The confinement and expansion of the plasmonic wave is dependent on the $\alpha$ . There is high confinement of field, if $\alpha$ is higher. On the other hand, smaller the $\alpha$ is responsible to the larger expansion of transverse fields into free space^18^. Hence, the grooved SSPP is linearly tapered. The phase constant of the tapered SSPP that can enable impedance matching with free space can be extracted using^28^,

7 $$\begin{aligned} \beta = k_{0} \times \frac{g}{p} \times tan(k_{0}h) \end{aligned}$$

According to Eq. (7), when the groove height is reduced to 0.2 mm, the phase constant is calculated to be approximately 1.04 rad/m. The proposed antenna design using this tapered groove SSPP is described in “Analysis and design of grooved SSPP antenna”(B).

To summarize, the proposed end-fire antenna requires a modulated grooved profile of SSPPs to support multiple modes, a total number of 16 grooves to maintain high gain and low SLLs, and a tapering grooved profile at the end of structure to match the impedance with free pace.

### Proposed antenna design

This section describes the overall geometry of the proposed linearly modulated grooved SSPPs based end-fire antenna.

**Fig. 9:**
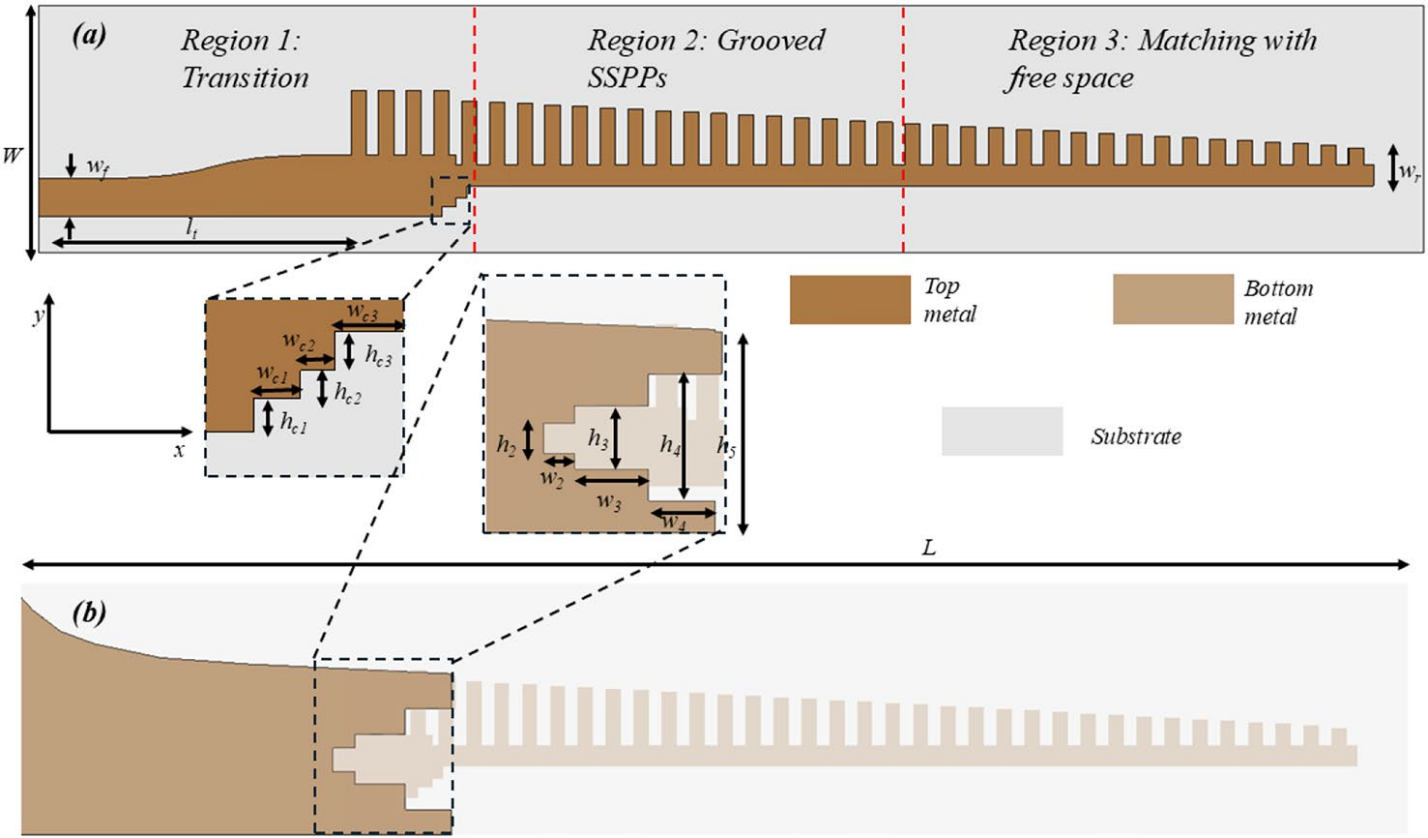
Configuration of the low-profile Grooved SSPPs based end-fire antenna (**a**) top-view; and (**b**) bottom view. Detailed dimensions (in mm): *W* = 9.86, *L* = 55, $l_{t}$ = 10, $w_{f}$ = 1.54, $w_{r}$ = 2.5, $h_{2}$ = 0.96, $h_{3}$ = 2, $h_{4}$ = 4, $h_{5}$ = 6.34, $w_{2}$ = 0.8, $w_{3}$ = 2, $w_{4}$ = 1.98, $w_{c1}$ = 0.58, $w_{c2}$ = 0.42, $w_{c3}$ = 0.64, $h_{c1}$ = 0.42, $h_{c2}$ = 0.34, and $h_{c3}$ = 0.50.

The proposed geometry of the modulated grooved SSPPs based end-fire antenna derived based on part A, Section II is shown in Fig. 9. The antenna is a single-layer structure printed on a Rogers RT-Duroid 5880 substrate with a dielectric constant, $\epsilon _{r}$= 2.2, loss tangent, $tan\delta$ = 0.0009 and thickness of 0.787 mm. Figure 9a shows the top layer of the proposed design. It is comprised of three sections: (i) 50 $\Omega$ microstrip feed line for antenna excitation; (ii) non-linearly tapered microstrip to SSPP transition, which converts the quasi-TEM modes in the microstrip line to SSPP modes and the broadband momentum matching; and (iii) linearly modulated grooved SSPP structure. Figure 9b shows the flaring and partial ground plane of the proposed design, which can help in matching the impedance between non-linear tapered transition and SSPPs. The detailed dimensions of overall geometry of the antenna are mentioned in Fig. 9. The antenna geometry is further explained with evolution of the ground plane and slots on the top-plane.

**Fig. 10:**
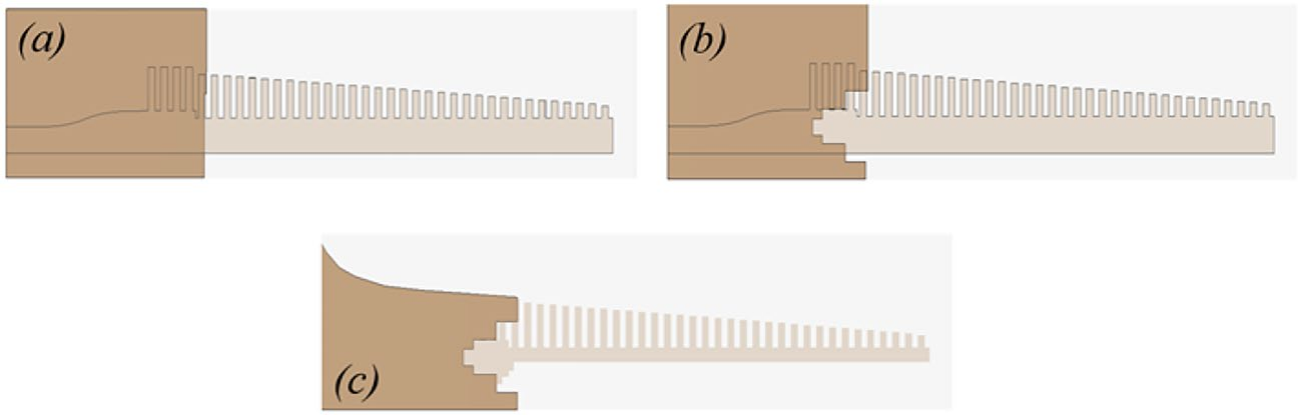
The evolution in the ground plane of the proposed design layout for SSPP based end-fire antenna with (**a**) Partial ground without slots; (**b**) ground with slots; (**c**) flaring in the ground. Dimensions marked in Fig. 9.

**Fig. 11:**
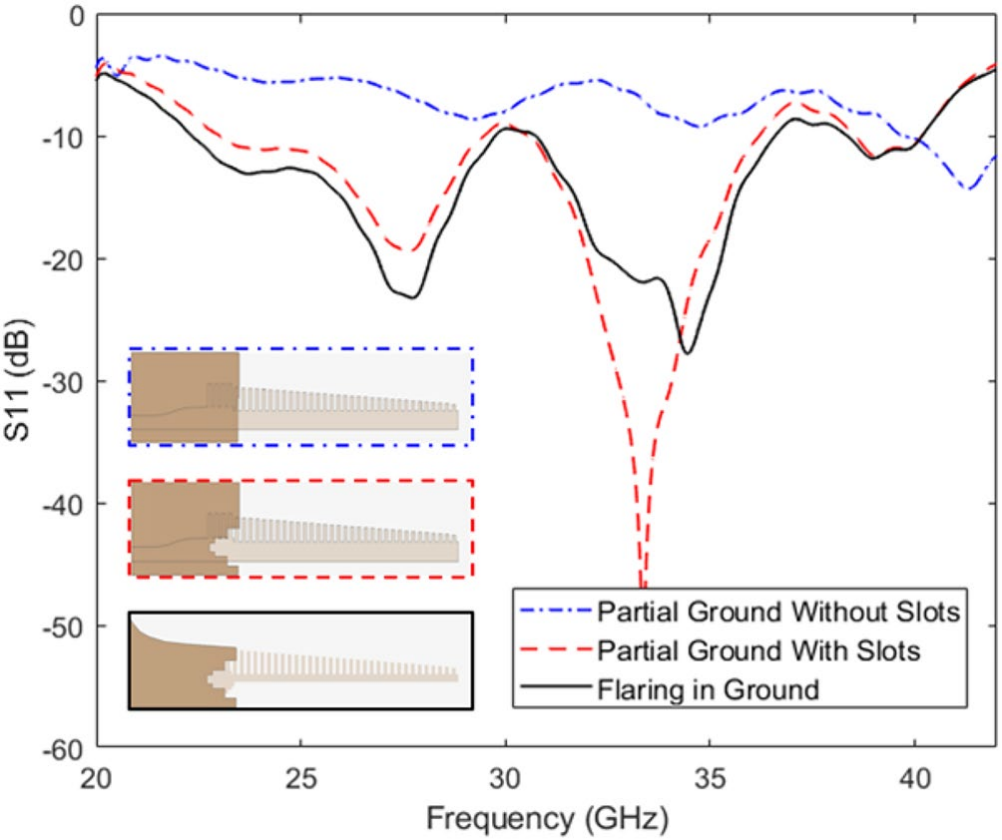
Simulated S11 of the proposed antenna with three different ground planes as defined in Figure 10.

The evolution of the ground plane is shown in Fig. 10. A discrete port was used to excite the antenna within the simulations. The reflection coefficients for each evolution stage are shown in Fig. 11. Initially we kept the partial ground plane that covers the feed line and transition between microstrip feed and grooved SSPPs. It gives a very poor -10 dB IBW in the operating region due to mismatch on impedance between tapered transition and grooved surface. To improve matching, we have created slots in Fig. 10a as shown in Fig. 10b, c.

The impact of slots in the ground plane is evaluated in terms of the reflection coefficient of the proposed antenna. It is clearly seen from Fig. 11 that slots in the ground plane help to improve the reflection coefficients and gives a -10 dB IBW between 23 GHz to 36 GHz. However, there is still a mismatch at 30 GHz due to transition region of mode 1 to mode 2. As an extension of the design, we incorporated a flaring ground profile which was a concept developed from^36^, to match the momentum and impedance of a plasmonic waveguide, and to achieve low loss transmission, see Fig. 10c. The flaring profile curve for the ground plane can be defined using Eq. (8)^34^,

8 $$\begin{aligned} y = C_{1} e^{Rx} + C_{2} \end{aligned}$$

where, $C_{1}$ and $C_{2}$ can be defined as,

9 $$\begin{aligned} C_{1}= & \frac{y_{2} - y_{1}}{e^{Rx_{2}} - e^{Rx_{1}}} \end{aligned}$$

10 $$\begin{aligned} C_{2}= & \frac{y_{2}e^{Rx_{2}} - y_{1}e^{Rx_{1}}}{e^{Rx_{2}} - e^{Rx_{1}}} \end{aligned}$$

Here, *R* is the radial value, and ($x_{1}$, $y_{1}$) and ($x_{2}$, $y_{2}$) are the start and end points of the profile curve.

**Fig. 12:**
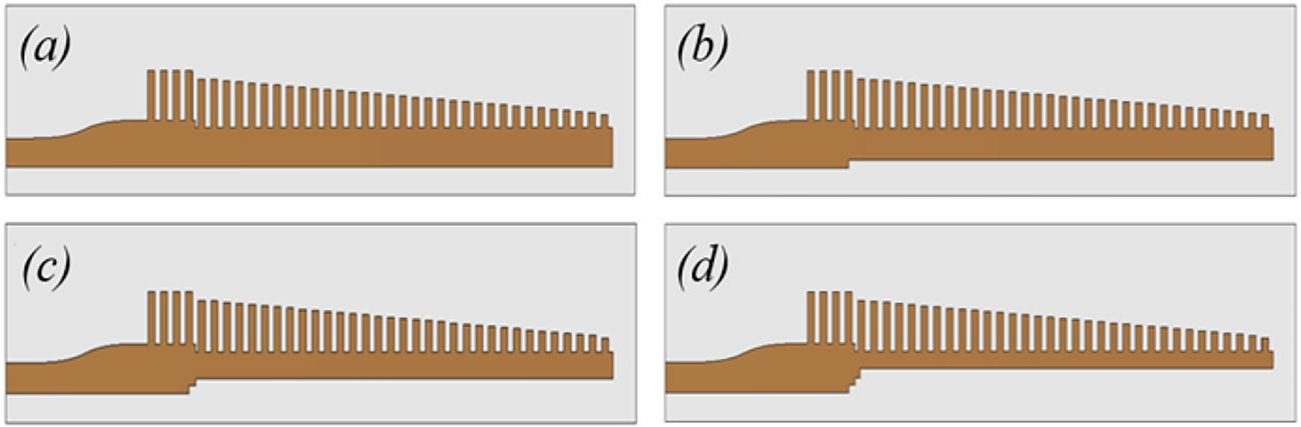
The evolution in the top-layer of the proposed design layout for SSPP based end-fire antenna with (**a**) no slots; (**b**) 1-step slot; (**c**) 2-step slot; and (**d**) 3-step slot. Dimensions marked in Fig. 9.

**Fig. 13:**
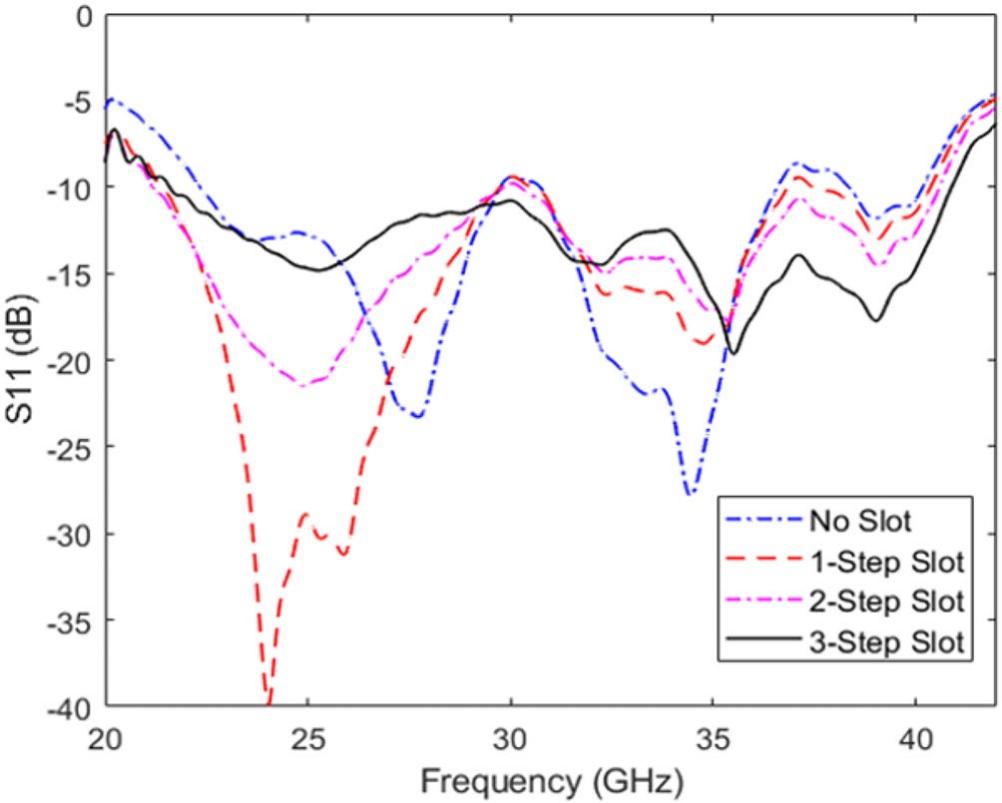
Simulated S11 of the proposed antenna with no slots; 1-step slot; 2-step slot; and 3-step slot.

To further improve the performance of antenna, rectangular stepped slots are etched out of the grooved SSPP profile on the top layer, see Fig. 12. These steps help to improve the impedance matching between the microstrip feedline and the groove SSPP transition. The simulated reflection coefficients for each stage are presented in Fig. 13. The 22 to 41 GHz band is maintained below -10 dB and ensures a good impedance match.

**Fig. 14:**
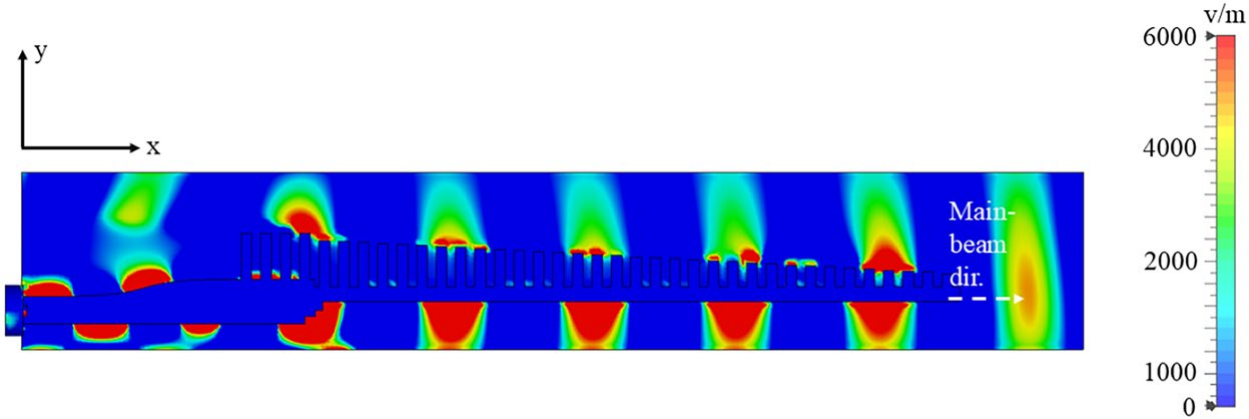
Simulated E-field plot for the optimized antenna design at 28 GHz..

**Fig. 15:**
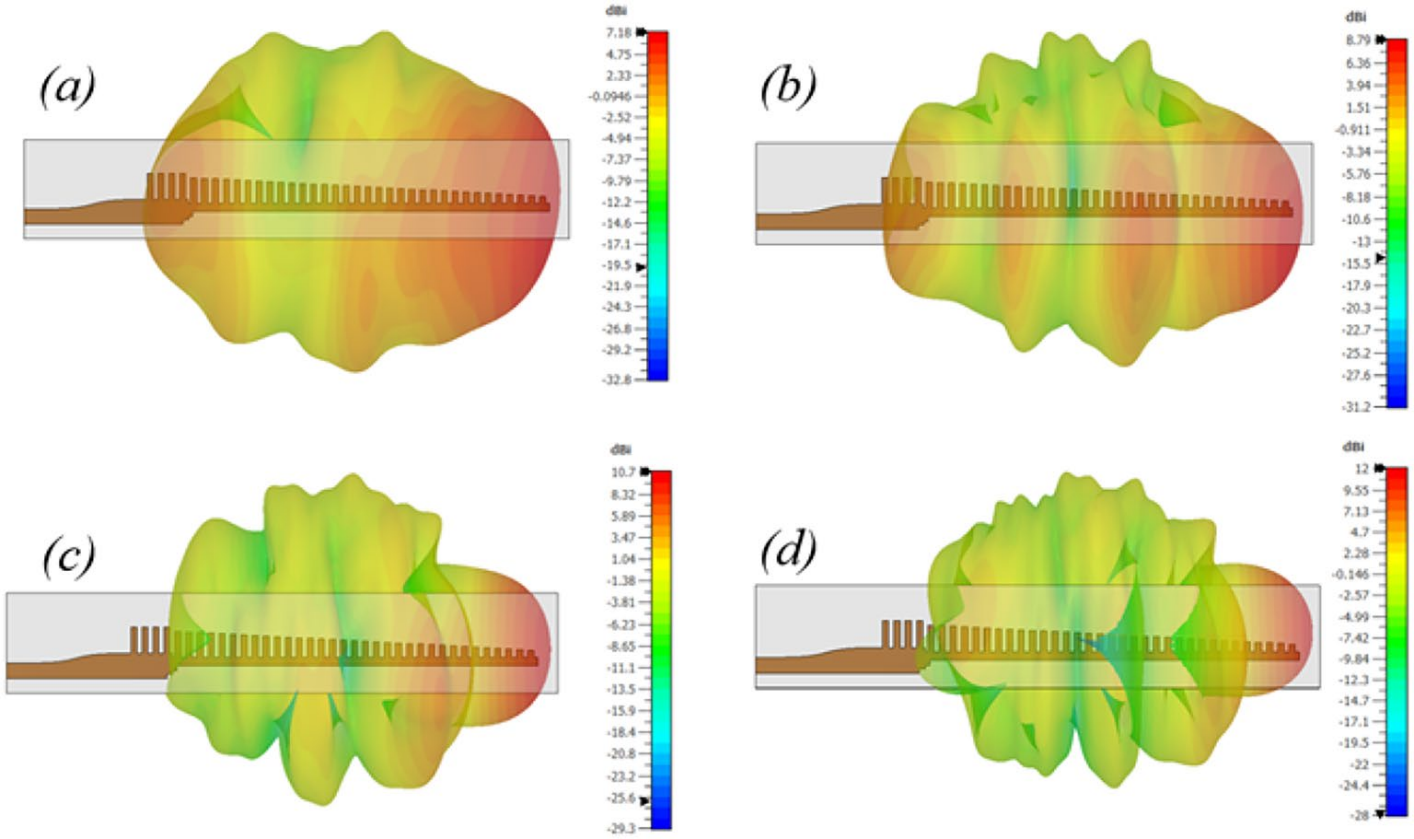
Simulated 3D radiation patterns at (**a**) 24 GHz; (**b**) 28 GHz; (**c**) 32 GHz; and (**d**) 36 GHz confirming end-fire beams generation.

The E-field distribution on the surface of the antenna is presented at 28 GHz in Fig. 14 confirms that the fields are well confined between the metal grooves and the dielectric laminate. The effective volume of this end-fire antenna is 55 mm $\times$ 12 mm $\times$ 0.51 mm. The main beam is maintained at end-fire throughout, and to further demonstrate that, simulated 3D radiation patterns at 24 GHz, 28 GHz, 32 GHz and 36 GHz are shown in Fig. 15.

**Fig. 16:**
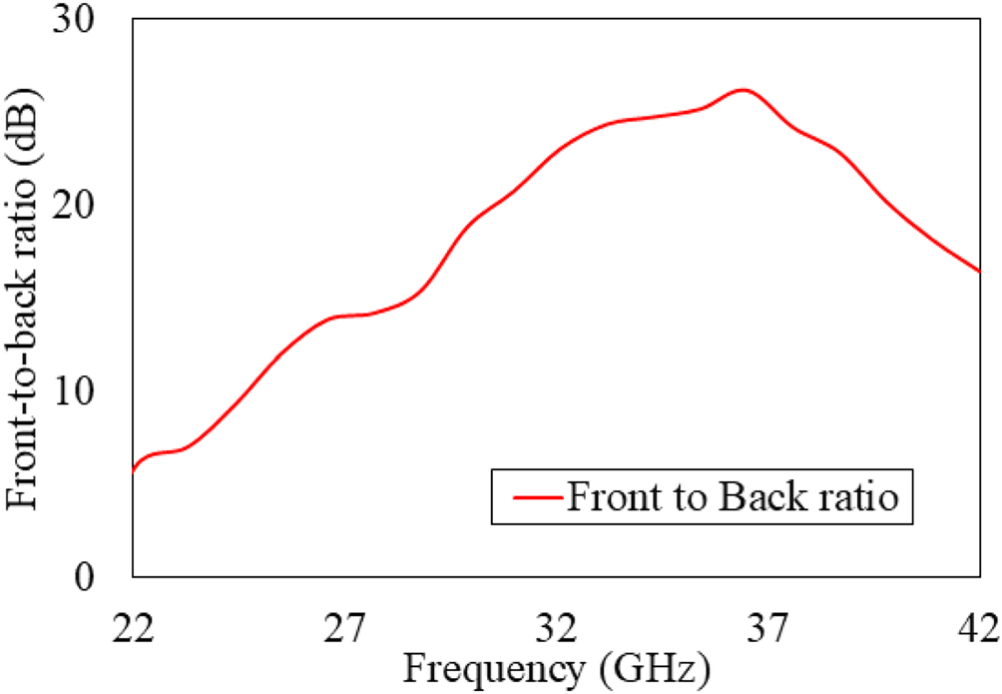
Simulated FBR of the linearly modulated grooved SSPP end-fire antenna.

**Fig. 17:**
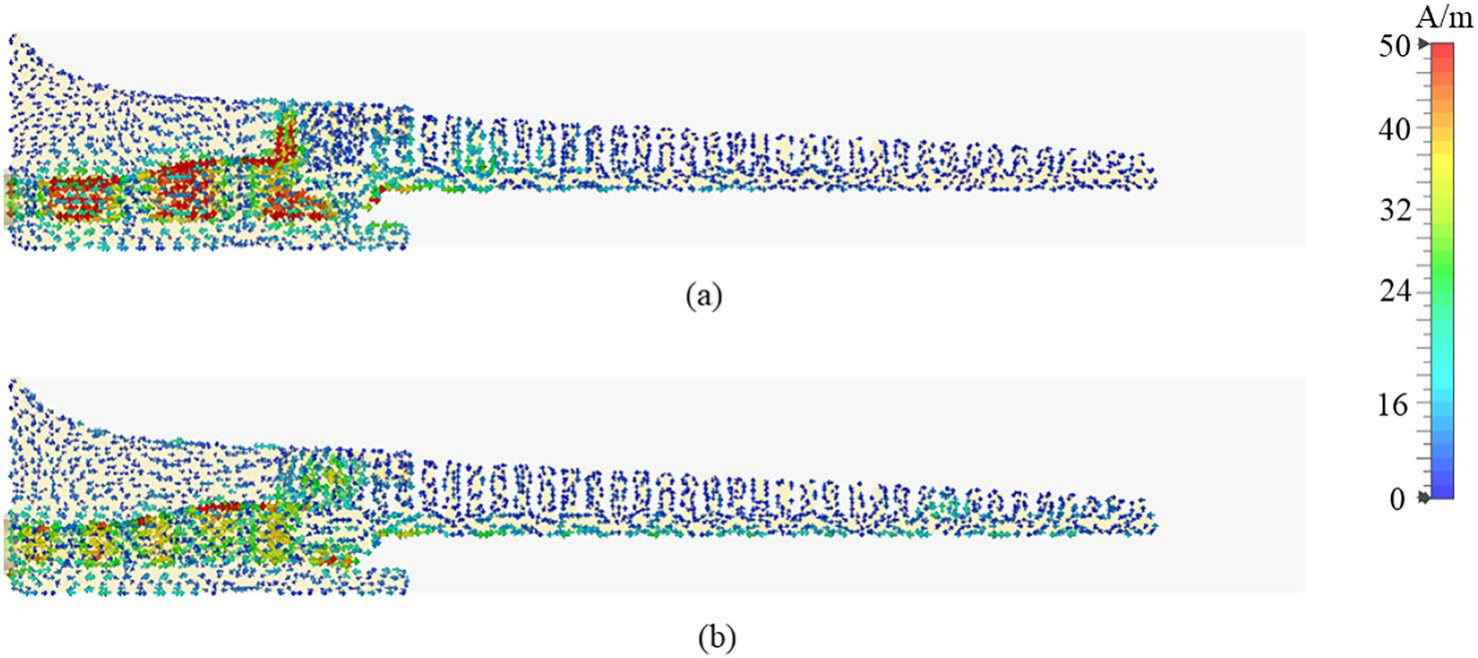
Simulated surface current distributions on the proposed antenna at (**a**) 23 GHz; and (**b**) 38 GHz.

The front-to-back-ratio (FBR) of the proposed antenna is illustrated in Fig. 16. It shows the proposed antenna has more than 10 dB above 25 GHz. The cause of this can be understood by reduction of gain at lower frequency range due to strong mutual coupling of the electric field between corrugations and reverse surface current flow due to higher order-modes, as shown in Fig. 17. Incorporation of electronic band gap (EBG) or frequency selective surface (FSS) structures between corrugations that will reduce inter-element coupling between corrugations and improve gain stability. However, due to smaller dimensions of EBG and FSS at mmWave frequencies often introduce fabrication complexity. In^37^, authors have proposed tilted corrugations technique to reduce the reverse surface current flow to stabilize the gain at lower frequency range. However, this typically leads to elevated side-lobe levels as well as offset the main beam from end-fire direction, which can increase interference—an undesirable trade-off for many applications.

Ultimately, achieving consistent gain performance involves balancing design complexity, fabrication feasibility, and system-level requirements. Depending on the intended application—whether stable gain or low interference is prioritized—the design can be further optimized accordingly.

## Fabrication and measurements

**Fig. 18:**
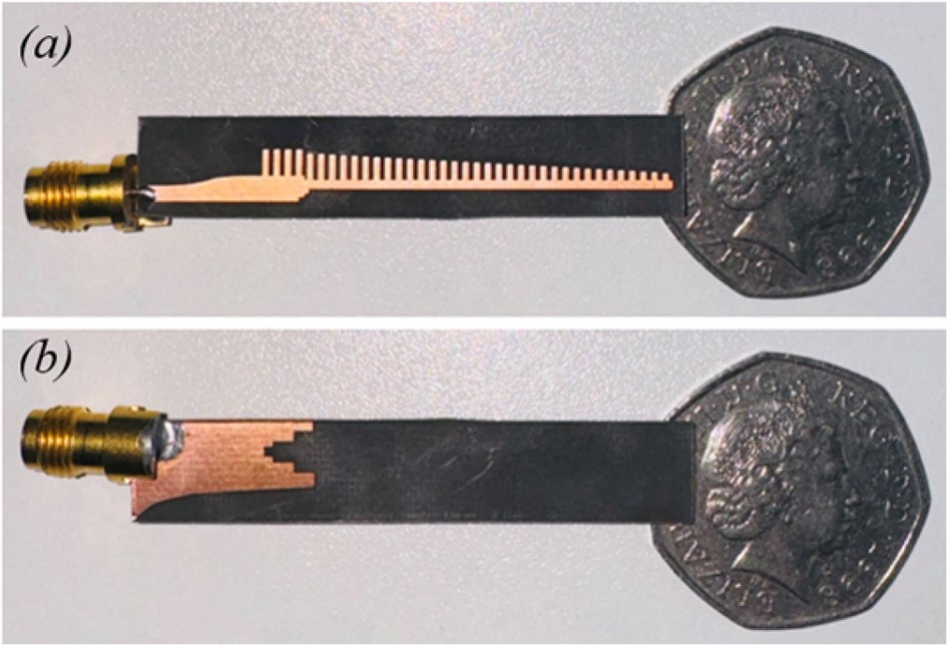
(**a**) Top; and (**b**) bottom view of the fabricated antenna.

The antenna was fabricated at Loughborough University using a standard photolithography process on a Rogers RT Duroid 5880 substrate. Figure 18 shows the fabricated prototype. To enable high frequency excitation, a 2.4 mm precision RF connector was soldered to the antenna’s feedline. This RF connector supports operation up to 50 GHz with an average insertion loss is less than 1.5 dB.

**Fig. 19:**
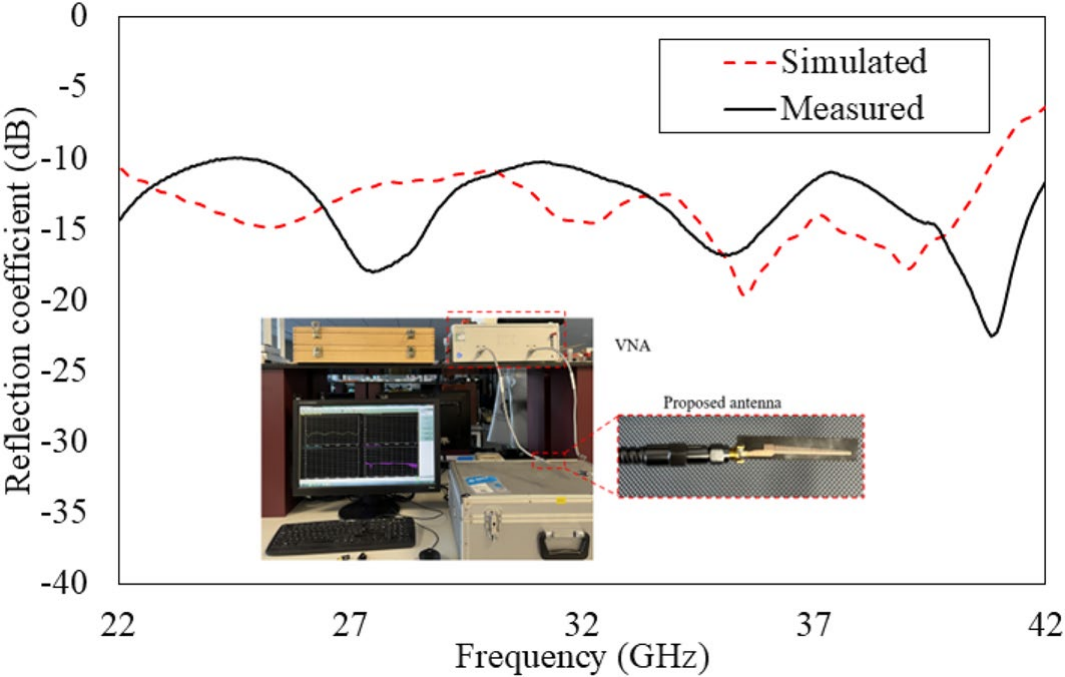
Simulated and measured S11 of the linearly modulated grooved SSPP end-fire antenna.

**Fig. 20:**
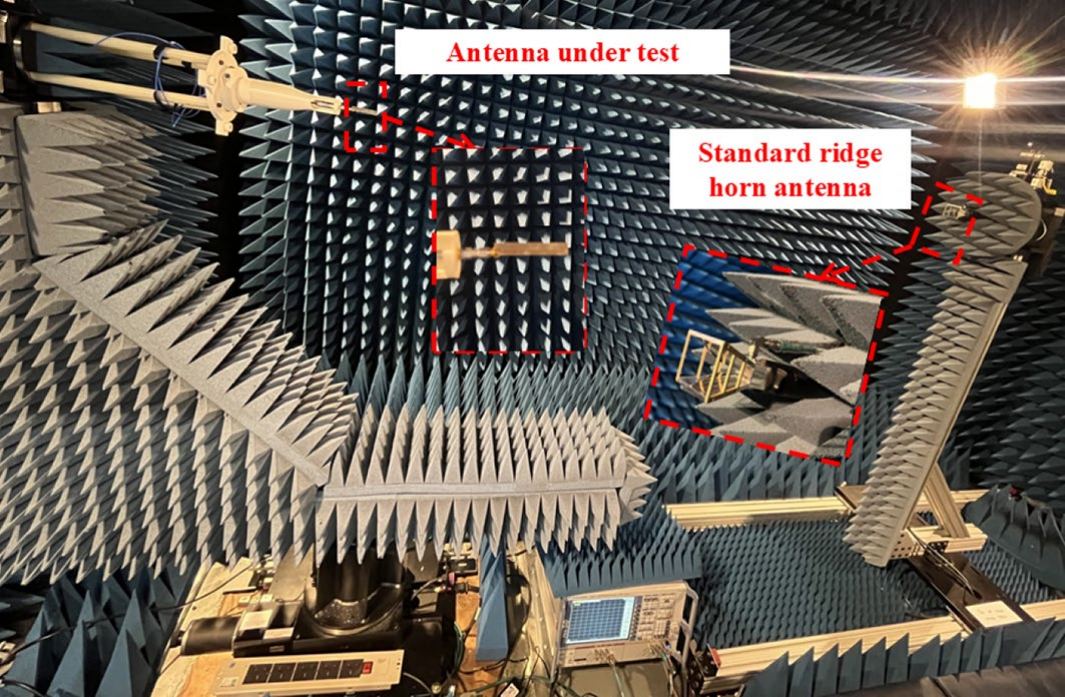
Test set-up for radiation patterns measurements of the linearly modulated grooved SSPP end-fire antenna.

The reflection coefficient ($\hbox {S}_{11}$) of the antenna was measured using an Anritsu MS46522B vector network analyzer, calibrated with SOLT (Short-Open-Load-Thru) method up to 43.5 GHz to ensure accurate de-embedding of cable and connector losses. Figure 19 presents the comparison of simulated and measured reflection coefficients of the proposed antenna. The antenna achieves a measured impedance bandwidth from 22 to 41 GHz, demonstrating strong agreement with simulations.

The slight mismatch in measured and simulated performance, particularly around 28 GHz, is attributed to the transition behavior between hybrid surface plasmon modes. In this region, the second-order SSPP mode has increased participation, and practical variations in etching, feed alignment, and metal thickness could lead to variations in modal excitation compared to idealized models. Future iterations could further mitigate this through more advanced lithography or laser direct structuring, which can offer higher dimensional accuracy.

To evaluate the radiation characteristics, the antenna was measured using a spherical near-field measurement system equipped with low-reflection absorbers optimized for the 0.7 to 50 GHz range, as shown in Fig. 20. The antenna under test (AUT) was mounted on a precision azimuth/elevation rotator, and a calibrated broadband standard ridge horn antenna, which has a gain > 10 dBi for frequency range of 10 to 40 GHz, was used as the reference antenna. The facility , developed by NSI-MI Technologies, utilizes spherical near-field scanning, and the far-field radiation patterns are extracted using proprietary near-field to far-field transformation algorithms.

**Fig. 21:**
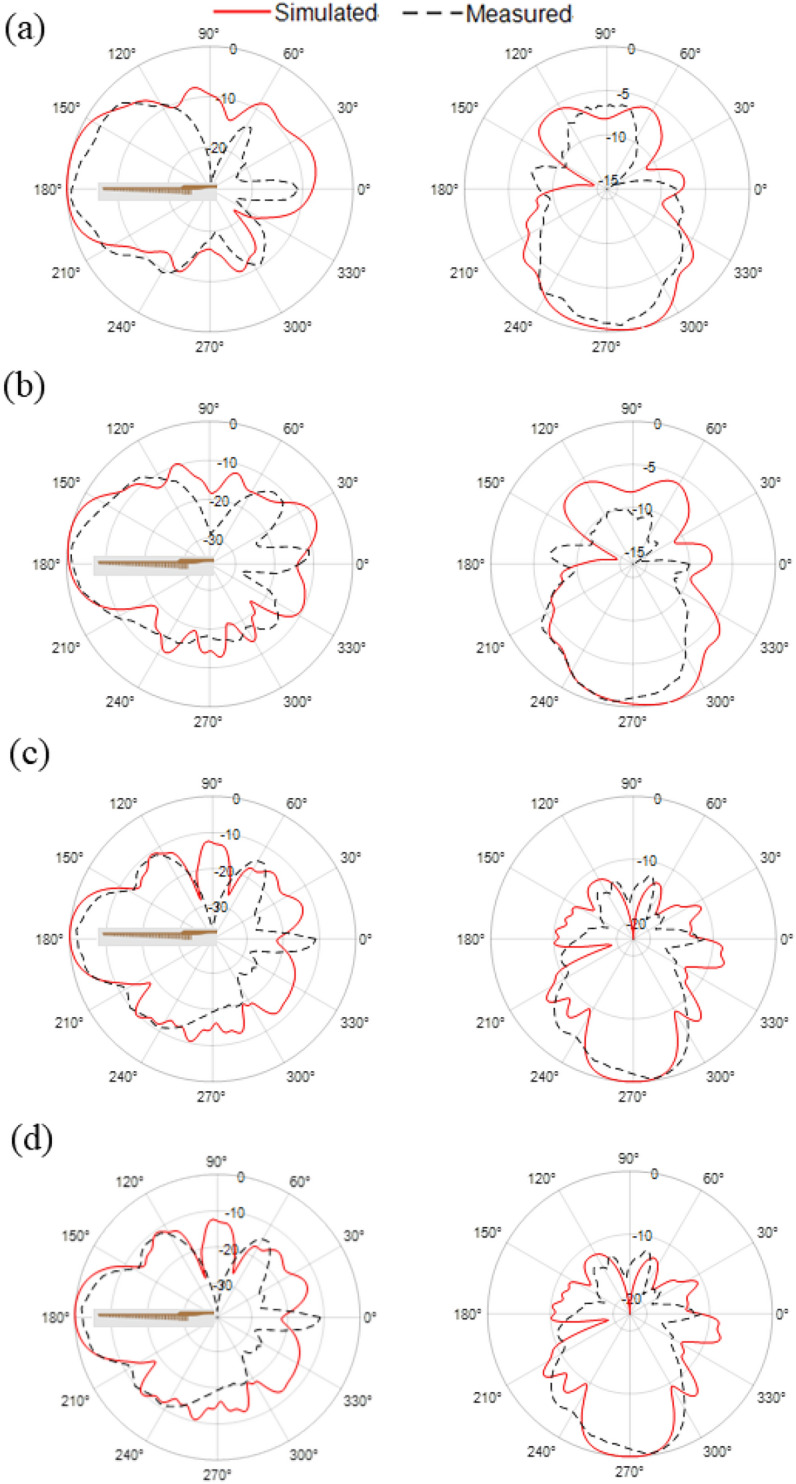
Simulated (red) and measured (black) radiation patterns in azimuth (left) and elevation (right) planes at (**a**) 24 GHz; (**b**) 28 GHz; (**c**) 32 GHz; and (**d**) 38 GHz.

The measured 2D radiation patterns are compared with the simulated in both azimuth and elevation planes at 24, 28, 32, and 38 GHz, which are shown in Fig. 21. The measured radiations patterns are well matched the simulated patterns, validating the antenna’s end-fire characteristics. The cross-polarized results were found to be below -20 dB, indicating strong polarization purity, and hence, are not shown here for brevity.

**Fig. 22:**
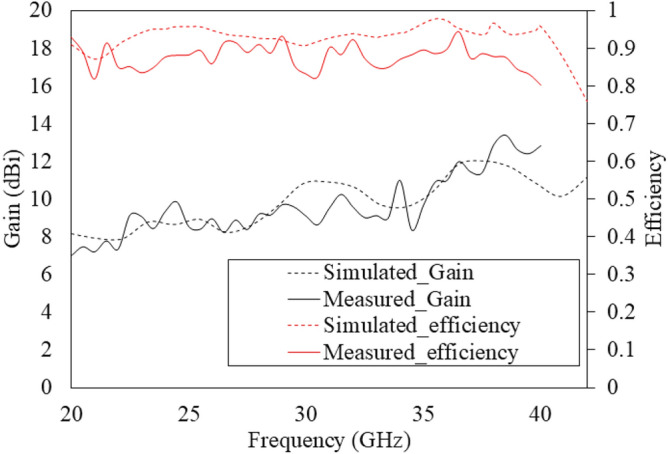
Simulated and measured gain (left axis) and efficiency (right axis) of the antenna.

The measured gain and total efficiency of the antenna were compared with simulations and are shown in Fig. 22, which closely follow the simulated trends across the 22 to 40 GHz range. The measured gain and efficiency is only shown until 40 GHz because of the limitation in the measurement capabilities. The agreement in simulated and measured results confirm that the fabricated antenna works throughout the frequency band.

The measured peak gain reaches approximately 13 dBi at 38 GHz, consistent with simulations. The average measured gain over the frequency is 10 dBi, with a minimum of 7.5 dBi. The minor discrepancies between the simulated and measured gain performances are attributed to substrate losses, surface roughness, fabrication tolerances, and soldering imperfections. They can certainly be improved with more precise fabrication methods. Benefiting from the low transmission loss of the grooved SSPP and the large equivalent radiating aperture, total measure efficiency better than 80 % (simulated efficiency is better than 90 %) is achieved within the operating frequency band.

Overall, the experimental results validate the antenna’s wideband and high-efficiency performance, confirming the efficacy of the design for future mmWave 5G applications.

**Table 1:** Comparison with other state-of-the art end-fire antennas.

Reference	Volume ($\lambda _{0}^{3}$)	Design type	Operating frequency (GHz)	Fractional BW (%)	Peak gain (dBi)	Average efficiency (%)
^15^	2.9 $\times$ 0.8 $\times$ 0.01	Corrugated SSPPs	7.5 − 8.5	12.5	9.2	90
^18^	6.5 $\times$ 2 $\times$ 0.02	Corrugated SSPPs	26.5 − 40	15	15	90
^28^	3.9 $\times$ 0.4 $\times$ 0.02	Corrugated SSPPs	8.5 − 17.5	69	14.4	72
^29^	8 $\times$ 0.4 $\times$ 0.02	Tilted Corrugated SSPPs	6.9 − 39.7	140.8	15.2	89
^34^	7.6 $\times$ 2.45 $\times$ 0.02	SSPPs	30 − 50	50	16	98
^38^	5 $\times$ 1.1 $\times$ 0.06	SSPPs	21 − 34	22.2	15.5	85
^39^	4.6 $\times$ 1.4 $\times$ 0.05	SSPPs $+$ Dielectric lens	7.9 − 13	49	14.8	80
^37^	4.82 $\times$ 0.6 $\times$ 0.01	SSPPs	12.9 − 34.6	60.1	17.3	88
^40^	9 $\times$ 1.8 $\times$ 0.03	SSPPs $+$ Phase shift stubs	26 − 30	10.7	15.2	81
^41^	9.2 $\times$ 2.6 $\times$ 0.02	SSPPs	30 − 50	50	17	90
^42^	9.36 $\times$ 1.8 $\times$ 0.1	SSPPs $+$ 2 layers	16 − 30	54.6	14.7	85
^43^	3.4 $\times$ 0.3 $\times$ 0.01	SSPPs	8 − 10	22.2	13.1	95
This work	5.5 $\times$ 1 $\times$ 0.05	Modulated corrugated SSPPs	22 − 41	62	13	92

## Conclusion

In this work, a novel wideband end-fire antenna based on a single-sided, linearly modulated grooved profile was presented. The antenna employs a groundless SSPP structure, offering advantages for high-density integration and packaging. Through careful optimization of the groove profile and ground-plane configuration, the proposed design achieves enhanced impedance matching and gain performance across a broad frequency range. The antenna is shown to achieve a peak gain of 13 dBi and a maximum efficiency of 90%, while operating over a wide bandwidth of 22 to 41 GHz, successfully producing a directive end-fire beam. The measured results are found to closely match with simulations with small variations contributed from fabrication tolerances. The overall volume of the proposed antenna is 5.5 $\lambda _{0}$ $\times$ 0.1 $\lambda _{0}$ $\times$ 0.05 $\lambda _{0}$, making it well-suited for emerging 5G mmWave applications. To highlight novelty of the design, a comparative performance analysis with existing state-of-the-art end-fire antennas is presented in Table 1. These results confirm the proposed antenna’s competitiveness in terms of bandwidth, gain, and integration potential.

## Data Availability

Data is provided within the manuscript.
